# Sleep quality and academic performance among medical students

**DOI:** 10.1192/j.eurpsy.2025.2336

**Published:** 2025-08-26

**Authors:** I. Baati, N. Regaieg, H. Trigui, M. Ben Jemaa, R. Masmoudi, I. Feki, F. Guermazi, J. Masmoudi

**Affiliations:** 1 Psychaitry A department; 2 Epidemiology department, University of Sfax, Faculty of medicine of Sfax, Sfax, Tunisia

## Abstract

**Introduction:**

Medical studies place significant academic pressure and high stress levels on students resulting in changes in their sleep patterns.and their academic performance, which are two keys of professional success. A hypothesis regarding a potential link between these two entitities could be proposed.

**Objectives:**

The objective of our study was to assess sleep quality and academic performance in a sample of students from the Faculty of Medicine of Sfax, Tunisia, as well as the link between these two entities.

**Methods:**

It was a cross-sectional, descriptive and analytical study, conducted using GOOGLE FORMS during February and March 2024, involving a sample of students from the Faculty of Medicine in Sfax, Tunisia. We used a questionnaire including an information sheet and two psychometric tests : the Pittsburgh Sleep Quality Index (PSQI) aiming to assess sleep quality over the past month and the Study Management and Academic Results Test (SMART) allowing the assessment of students’ attitudes towards their studies and academic performance based on four dimensions : “Academic Competence”, “Test Competence”, “Time Management” and “Strategic Studying”.

**Results:**

Our study involved 154 participants with a sex ratio (M/F) of 0.54 and a median age of 22 years (IQR = [20 – 23 years]).

The median PSQI score was 6 (IQR = [3 – 9]). Using a threshold value of 5, we found that 86 students had poor sleep, resulting in a prevalence of 55.8%.

Median scores of the four dimensions were 3.4 (IQR = [3 – 3.8]) for the “Academic Competence”, 2.8 (IQR = [2.2 – 3.2]) for the “Test Competence”, 2.4 (IQR = [2 – 3]) for the “Time Management” and 3.2 (IQR = [2 .8 – 3.6]) for the “Strategic Studying”.

By conducting a bivariate analysis, we found that “Academic Competence” and “Time Management” dimensions were significantly better among students with good sleep quality. In contrast, the dimensions “Test Competence” and “Strategic Studying” were not statistically associated with sleep quality (Table 1).

**
Table 1: Associations between the sleep quality and the Study Management and Academic Results Test dimensions**

**Table:** 

	Academic Competence	Test Competence	Time Management	Strategic Studying
**Sleep quality**				
Good	3.6 (3.2 – 4)	2.8 (2.4 – 3.3)	2.6 (2.2 – 3)	3.2 (2.8 – 3.6)
Poor	3.4 (2.8 – 3.6)	2.8 (2.2 – 3.2)	2.4 (1.8 – 2.8)	3.2 (2.8 – 3.6)
p	**0.01**	0.5	**0.02**	0.7

**Conclusions:**

Our study revealed that more than half of the medical students suffer from poor sleep quality. The analysis of academic performance revealed that the most affected dimensions were the test competence and the time managemement. The lack of sleep among these students had detrimental consequences on their academic performance. Therefore, it is important to encourage good sleep hygiene to enhance both well-being and academic performance in medical students. Additionally, providing balanced study resources, offering therapy and counseling services, and promoting stress management strategies are key to optimizing academic success.

**Disclosure of Interest:**

None Declared

